# Increased stable integration efficiency in CHO cells through enhanced nuclear localization of Bxb1 serine integrase

**DOI:** 10.1186/s12896-024-00871-4

**Published:** 2024-06-26

**Authors:** Olli Huhtinen, Stuart Prince, Urpo Lamminmäki, Rune Salbo, Antti Kulmala

**Affiliations:** 1 Protein & Antibody Engineering, Orion Corporation, Turku, Finland; 2 Department of Life Technologies, University of Turku, Turku, Finland; 3 MediCity Research Laboratory, University of Turku, Turku, Finland; 4 Institute of Biomedicine, University of Turku, Turku, Finland

**Keywords:** Bxb1 serine integrase, Nuclear localization signal, Mammalian display

## Abstract

**Background:**

Mammalian display is an appealing technology for therapeutic antibody development. Despite the advantages of mammalian display, such as full-length IgG display with mammalian glycosylation and its inherent ability to select antibodies with good biophysical properties, the restricted library size and large culture volumes remain challenges. Bxb1 serine integrase is commonly used for the stable genomic integration of antibody genes into mammalian cells, but presently lacks the efficiency required for the display of large mammalian display libraries. To increase the Bxb1 integrase-mediated stable integration efficiency, our study investigates factors that potentially affect the nuclear localization of Bxb1 integrase.

**Methods:**

In an attempt to enhance Bxb1 serine integrase-mediated integration efficiency, we fused various nuclear localization signals (NLS) to the N- and C-termini of the integrase. Concurrently, we co-expressed multiple proteins associated with nuclear transport to assess their impact on the stable integration efficiency of green fluorescent protein (GFP)-encoding DNA and an antibody display cassette into the genome of Chinese hamster ovary (CHO) cells containing a landing pad for Bxb1 integrase-mediated integration.

**Results:**

The nucleoplasmin NLS from *Xenopus laevis*, when fused to the C-terminus of Bxb1 integrase, demonstrated the highest enhancement in stable integration efficiency among the tested NLS fusions, exhibiting over a 6-fold improvement compared to Bxb1 integrase lacking an NLS fusion. Subsequent additions of extra NLS fusions to the Bxb1 integrase revealed an additional 131% enhancement in stable integration efficiency with the inclusion of two copies of C-terminal nucleoplasmin NLS fusions. Further improvement was achieved by co-expressing the Ran GTPase-activating protein (RanGAP). Finally, to validate the applicability of these findings to more complex proteins, the DNA encoding the membrane-bound clinical antibody abrilumab was stably integrated into the genome of CHO cells using Bxb1 integrase with two copies of C-terminal nucleoplasmin NLS fusions and co-expression of RanGAP. This approach demonstrated over 14-fold increase in integration efficiency compared to Bxb1 integrase lacking an NLS fusion.

**Conclusions:**

This study demonstrates that optimizing the NLS sequence fusion for Bxb1 integrase significantly enhances the stable genomic integration efficiency. These findings provide a practical approach for constructing larger libraries in mammalian cells through the stable integration of genes into a genomic landing pad.

## Background

Various display platforms have become the cornerstone of modern antibody and protein engineering. Among these mammalian display possesses beneficial characteristics to produce antibodies with good biophysical properties [1, 2]. In addition, mammalian display has the ability to display full-length IgGs with mammalian glycosylation, making it an appealing method for therapeutic antibody discovery in comparison to other platforms such as phage, bacterial, and yeast display. Optimal construction of mammalian display libraries requires the stable integration of each library variant into a predetermined genomic locus, ensuring that each cell contains only one variant. This approach facilitates multiple selection rounds, allows for comparison of expression levels among library variants, and mitigates the enrichment of undesired “passenger” variants during selection [3]. A current limitation in mammalian display is the restricted library size and the large culture volumes with large libraries. While viral vectors are efficient for stable gene transfection into mammalian cells [4–6], they pose the risk of random gene insertion and the potential for multiple gene copies within a single cell.

To attain stable, single-copy gene integration, a “landing pad” can be employed. This landing pad is initially integrated into the organism’s genome, and the gene of interest is subsequently integrated into the landing pad using serine or tyrosine integrases [7–11]. Among various integrases, the serine integrase from Mycobacteriophage Bxb1 stands out as one of the most widely used for the stable integration of genes [7–12] owing to its exceptional accuracy and efficiency [13]. Bxb1 integrase catalyzes site-specific recombination between *attP* and *attB* sites, enabling stable integration of a gene of interest into the genome when a vector carrying the gene of interest contains an *attP* site and the landing pad possesses an *attB* site [12, 14]. In a scenario where a single attB site is present in the genome and a single attP site is present in the vector, complete plasmid integration into the genome occurs. Alternatively, recombinase-mediated cassette exchange (RMCE) allows for the selective integration of the gene of interest, excluding the vector. This is achieved when a genomic segment is flanked by two distinct attB sites, and the gene of interest within the vector is flanked by two distinct attP sites [15, 16].

The stable integration of a gene into an organism’s genome is a relatively rare event [17]. Various approaches have been investigated to improve the efficiency of Bxb1 integrase-mediated stable gene integration, including continuous expression of Bxb1 integrase by integrating it into the landing pad [8], and optimization of transfection [7]. For Bxb1 integrase to perform its function, it has to enter the cell’s nucleus. Proteins with a molecular weight below 40 kDa can enter the nucleus through nuclear pore complexes (NPCs) via passive diffusion. However, larger proteins, such as Bxb1 integrase (approximately 56 kDa) [14], require a nuclear localization signal (NLS) for efficient nuclear entry [18, 19]. NLSs are signal sequences that direct proteins into the nucleus via an active translocation pathway. Proteins equipped with NLS are actively translocated into the nucleus by importin α and importin β. Importin α interacts with importin β through its importin-β-binding (IBB) domain, which also serves as a regulative domain for NLS binding. In the absence of interaction between importin α and importin β, the IBB domain occupies the NLS binding site, preventing the binding of NLS to the site. Upon interaction between importin α and importin β, importin β binds to the IBB domain, allowing NLS to bind to the site. The resulting ternary complex is then translocated into the nucleus. In the nucleus, the complex is dissociated upon binding of Ran-GTP to importin β, which also initiates recycling of importins to the cytoplasm. Exportin CAS-Ran-GTP translocates importin α back to the nucleus, after which importin α is released from the complex by Ran GTPase-activating protein (RanGAP), leading to the conversion of Ran-GTP to Ran-GDP. Ran-GDP is converted back to the active Ran-GTP form by Regulator of chromosome condensation 1 (RCC1) protein [20–22].

Although the translocation process plays a pivotal role in Bxb1 integrase function in mammalian cells, a systematic study examining the impact of various translocation pathway elements on Bxb1 integrase-mediated stable integration efficiency is lacking. This prompted us to investigate the effect of different NLS types and proteins involved in nuclear translocation process on Bxb1 integrase-mediated stable integration efficiency in CHO cells containing a landing pad for the stable genomic integration of genes. First we tested the fusion of two monopartite cNLSs (Myc proto-oncogene protein NLS and Simian Virus 40 NLS) and two bipartite NLSs (Transcription factor EGL-13 NLS and nucleoplasmin NLS from *Xenopus laevis*) in different configurations to Bxb1 integrase. Additionally, the potential role of nuclear translocation-associated proteins to Bxb1 integrase-mediated genomic integration were explored by co-expression of importin α, importin β, RanGTP, RCC1 and RanGAP. We discovered that the stable integration efficiency by Bxb1 integrase is influenced not only by the type of NLS but also by the number and location of the NLS. Furthermore, co-expression of RanGAP was observed to have a positive effect on the stable integration efficiency. Our findings demonstrate the potential to substantially enhance Bxb1 integrase-mediated stable integration efficiency in CHO cells through the incorporation of an optimized NLS configuration for Bxb1 integrase and the augmentation of nuclear transport via co-expression of RanGAP.

## Materials and methods

### Mammalian display cell line and vector construction

Mammalian display CHO cell line, CHO-LP, was generated by integrating a Bxb1 landing pad (LP) into the genome of Flp-In CHO cell line (Invitrogen, RRID: CVCL_U424), described earlier [11]. The Bxb1 LP contained cytomegalovirus (CMV) promoter-driven membrane-anchored mouse IgG2a Fc and blasticidin S deaminase (BSD), flanked by AttB and AttBm sites, enabling directional RMCE. In this study, we used a promoterless Bxb1 targeting vector, pBxb1-TV, encoding membrane anchored EGFP (pBxb1-TV-GFP) or abrilumab (pBxb1-TV-Abrilumab) and Bxb1 integrase (Uniprot ID: Q9B086) expression vector, pBxb1-EV, for evaluating the stable transfection efficiency, as described previously [11]. The pBxb1-TV-GFP consisted of DNA encoding membrane anchored EGFP and puromycin N-acetyltransferase, flanked by AttP and AttPm sites for RMCE. In the pBxb1-TV-Abrilumab, the EGFP encoding DNA was exchanged to DNA encoding abrilumab light chain, furin cleavage site, T2A peptide, IGHV3 signal peptide and abrilumab heavy chain, which was synthesized and cloned into the pBxb1-TV by Twist Biosciences. The NLS variants (Myc proto-oncogene NLS, Uniprot ID: P01106, simian virus 40 large T antigen NLS, Uniprot ID: P03070, transcription factor EGL-13 NLS, Uniprot ID: Q23045 and nucleoplasmin NLS from *Xenopus laevis*, Uniprot ID: P05221) were synthesized by GeneArt (Thermo Fisher) and cloned into the N-terminus and C-terminus of Bxb1 by using BamHI-AvaI and PshAI-SphI restriction sites in the pBxb1-EV vector, respectively. The DNA encoding two copies of the nucleoplasmin NLS and a nuclear transport protein (importin α, Uniprot ID: P52293, importin β, Uniprot ID: P70168, RanGTP, Uniprot ID: P62827, RCC1, Uniprot ID: Q8VE37 or RanGAP, Uniprot ID: A0A2R8W753), separated by furin cleavage site and T2A peptide were synthesized by GeneArt and cloned into the C-terminus of Bxb1 by using PshAI-SphI restriction sites in the pBxb1-EV vector.

### Cell culture and transfections

The CHO-LP cells were cultured in Ham’s F-12 Nutrient Mix (Gibco) supplemented with 2 mM GlutaMAX (Gibco) and 10% fetal bovine serum (Sigma-Aldrich) (hereafter referred as F-12 medium) at + 37 °C, 5% CO_2_. The day before transfection, the wells of 6-well plates (Biocoat poly-D-Lysine cellware, Corning) containing 2 ml of F-12 medium were seeded with CHO-LP cells (0.4 × 10^6^ cells/well), and the cells were grown overnight. The next day the CHO-LP cells were co-transfected with Bxb1–NLS fusion variant in pBxb1-EV vector (1.25 µg) and pBxb1-TV-GFP vector (1.25 µg) using Lipofectamine 3000 reagent according to the manufacturer’s recommendations (Thermo Fisher Scientific). Non-transfected cells and cells transfected only with pBxb1-TV-GFP (1.25 µg) were used as negative and transient expression controls, respectively, to make the gating in flow cytometry. Three days post-transfection, the cells were passaged. First, the old medium was discarded, and the cells were washed with 1 ml of DPBS (without calcium and magnesium) (Thermo Fisher Scientific) and 1 ml of 0.05% Trypsin-EDTA was added to the wells. The trypsination reaction was incubated at 37 °C with 5% CO_2_ for 6 min. After the incubation, 1 ml of F-12 medium was added to the wells to stop the trypsination reaction. Subsequently, the cells were split in 1:10 ratio to new 6-well plates containing fresh F-12 medium. The cells were then further cultured for three days before the flow cytometry analysis. In the experiments where Bxb1-NPLCx2 and nuclear translocation-associated proteins were co-expressed, the DNA amounts for Bxb1 lacking NLS fusion were 1.25 µg of pBxb1-EV and 1.25 µg of pBxb1-TV-GFP or pBxb1-TV-Abrilumab. The Bxb1 variants containing the nuclear translocation-associated protein gene were transfected using the same molar amount of the pBxb1-EV plasmid as the Bxb1 lacking the NLS fusion.

### Flow cytometry analysis

The culture medium was discarded, and the cells were washed with 1 ml of DPBS, and 1 ml of Corning Cellstripper Dissociation Reagent (Thermo Fisher Scientific) was added to the wells, and subsequently the cells were incubated at 37 °C with 5% CO_2_ for 20 min. The dissociation reactions were stopped by adding 1 ml of F-12 medium. The dissociated cells were then pipetted to 15 ml Falcon tubes (Thermo Fisher Scientific). The cells were pelleted by centrifugation (1000 x g, 5 min, + 4 °C), the supernatant was discarded, and the cells were resuspended in 200 µl of cold EasySep Buffer (Stemcell Technologies, Cambridge, UK). The abrilumab-transfected cells were stained with PE anti-human IgG Fc antibody (BioLegend, USA) using 5 µl of the antibody in 100 µl of cells in EasySep Buffer, incubating them 30 min on ice, after which they were washed by centrifugation and resuspended to 200 µl of cold EasySep Buffer. Subsequently, the resuspended cells were pipetted to U-bottom 96-well plate (Micro test plate 96 well, Sarstedt, Nümbrecht, Germany), which was kept on ice. The stable integration efficiency was assessed by measuring the percentage of GFP positive or PE positive cells with BD Accuri Flow Cytometer (BD Biosciences). The obtained flow cytometry data was analyzed with FlowJo software (BD Biosciences). To enable comparison between transfections done on separate days, the stable integration efficiency of each Bxb1-NLS variant was normalized to Bxb1 integrase lacking the NLS fusion.

### Statistical analysis

Statistical analyses were done using GraphPad Prism 9 (GraphPad, San Diego, USA) and Microsoft Excel. Student’s T-test with assumed equal variances was used to test for statistically significant difference between the Bxb1 NLS variants. The threshold for statistical significance (alpha level) was 0.05.

## Results

### Nucleoplasmin nuclear localization signal fusion in Bxb1 integrase substantially enhances stable integration efficiency

**Fig. 1:**
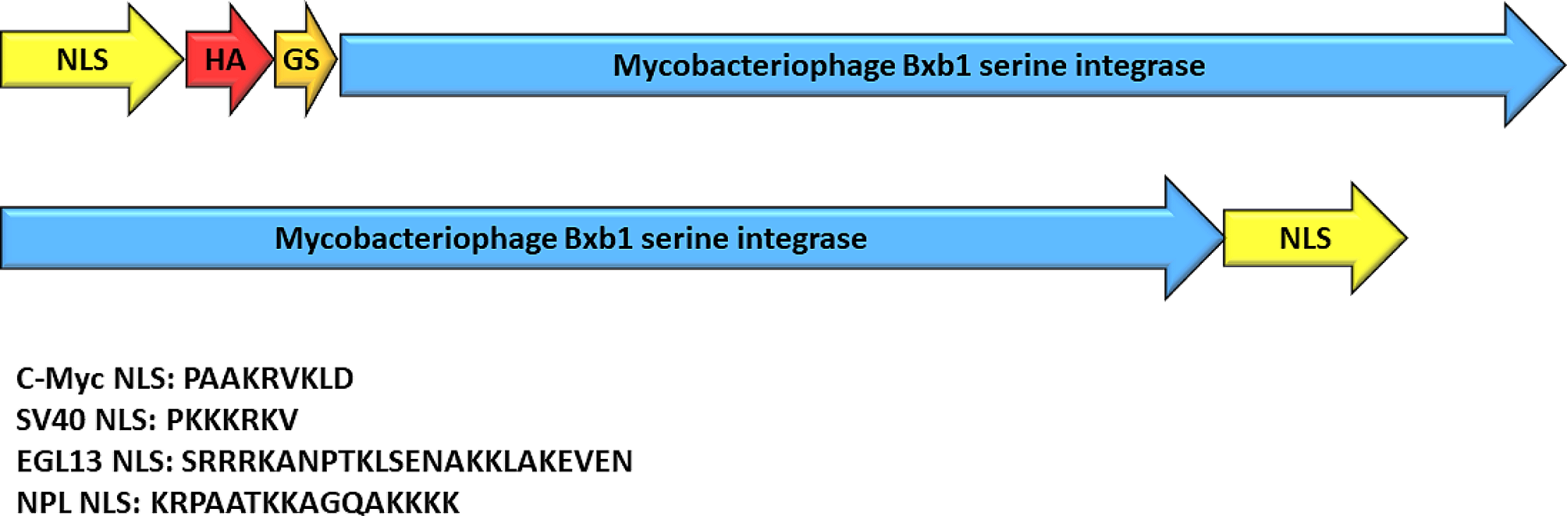
Mycobacteriophage Bxb1 serine integrase with N-terminal and C-terminal nuclear localization signal fusions (NLS). Upper construct illustrates Mycobacteriophage Bxb1 serine integrase with N-terminal NLS fusion. N-terminal NLS (yellow) and Mycobacteriophage Bxb1 serine integrase gene (blue) was separated by a GS linker (orange) and human influenza hemagglutinin (HA) epitope tag (red). Lower construct illustrates Mycobacteriophage Bxb1 serine integrase with C-terminal NLS. Amino acid sequences of the NLSs are shown below the constructs

A Chinese hamster ovary cell line with a single copy of a Bxb1 landing pad (CHO-LP) has been used for the precise genomic integration of antibody genes, enabling the screening of large antibody libraries [11]. Genomic integration is achieved through the co-transfection of a gene of interest in the Bxb1 targeting vector (pBxb1-TV) and a Bxb1 integrase expression vector (pBxb1-EV). In this study, we demonstrate the enhancement of Bxb1 integrase-mediated genomic integration efficiency by fusing the integrase with various nuclear localization signal (NLS) sequences. Bxb1 integrase–NLS variants, incorporating Myc proto-oncogene NLS (c-Myc NLS), simian virus 40 large T antigen NLS (SV40 NLS), transcription factor EGL-13 NLS (EGL-13 NLS), or nucleoplasmin NLS from *Xenopus laevis* (NPL NLS) at the N- or C-terminus of the Bxb1 integrase gene, were generated (Fig. 1). The variants were named Bxb1–c-MycN/C, Bxb1–SV40N/C, Bxb1–EGL-13 N/C, and Bxb1–NPLN/C, with N or C indicating the position of the NLS fusion.

The impact of the NLS fusion to the Bxb1 integrase was investigated by co-transfecting CHO-LP cells with pBxb1-TV-GFP along with different Bxb1–NLS variants in a 1:1 vector ratio by weight. Following transfection, cells were cultured for six days without antibiotic selection, and the stable integration efficiency of each Bxb1–NLS variant was evaluated by quantifying the percentage of GFP-positive cells using flow cytometry. Non-transfected cells and cells transfected only with pBxb1-TV-GFP vector (transient expression control) were used as gating controls. To minimize transfection-related variability and to enable the comparison between individual experiments, the stable integration efficiency of each Bxb1–NLS variant was normalized to the stable integration efficiency of Bxb1 integrase lacking the NLS fusion.

**Fig. 2:**
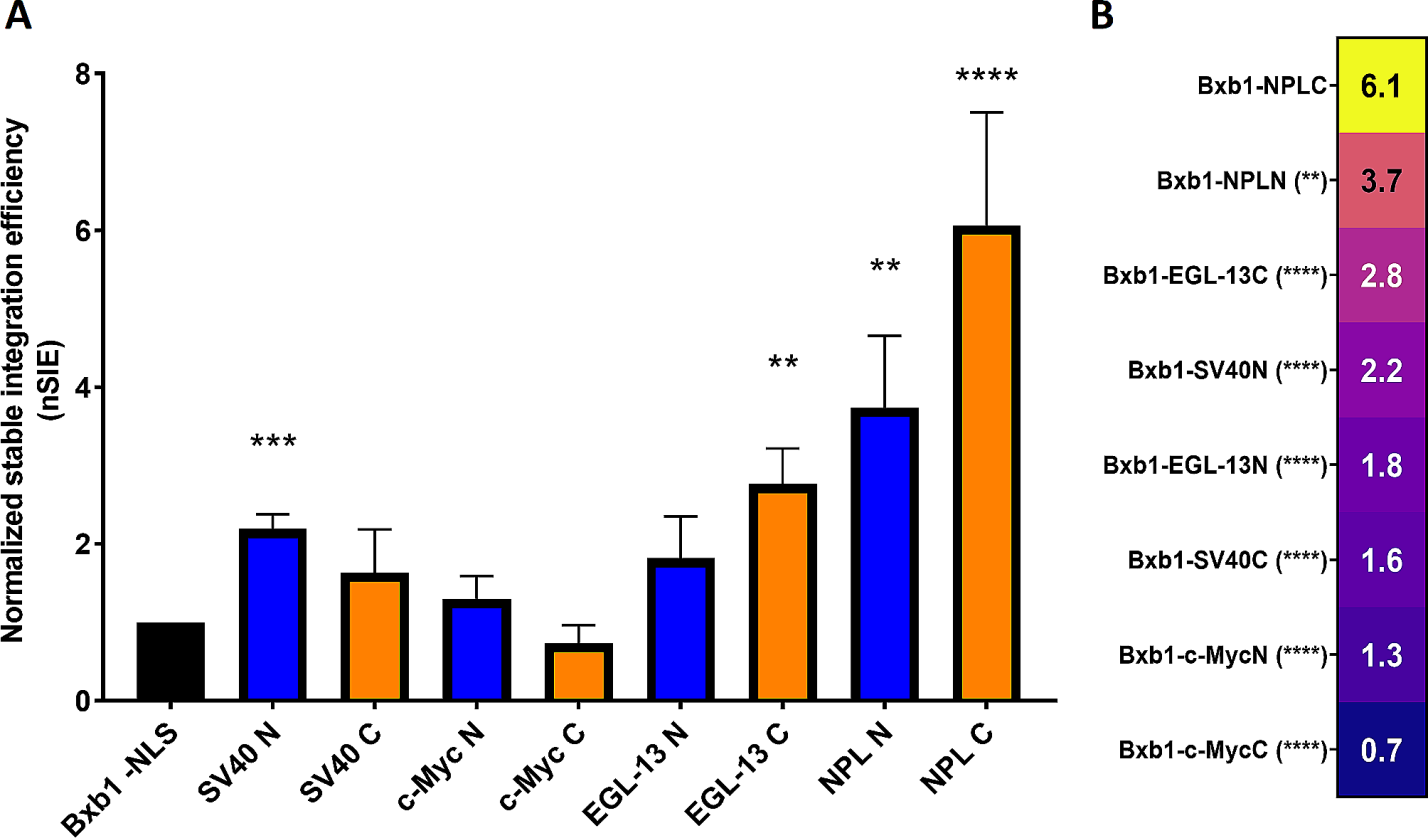
Normalized stable integration efficiencies (nSIE) of the tested Bxb1–NLS variants. (**A**) The graph shows the average nSIEs of Bxb1–NLS variants carrying either N-terminal (blue columns) or C-terminal (orange columns) NLS fusion. The error bars represent standard deviation of three independent experiments (except NPLC, *n* = 6). Asterisks denote statistically significant difference to Bxb1 without NLS (Bxb1 -NLS) (**B**) Bxb1–NLS variants sorted according to nSIE. Asterisks denote statistically significant difference to Bxb1–NPLC. (**) *p* < 0.01; (***) *p* < 0.001; (****) *p* < 0.0001

Among the tested Bxb1–NLS variants, Bxb1–NPLC demonstrated the highest normalized stable integration efficiency (nSIE) (Fig. 2) exhibiting more than sixfold increase in nSIE compared to Bxb1 integrase without the NLS fusion (*p =* 0.0006). The second-best variant was Bxb1–NPLN, which increased nSIE by 3.7-fold compared to Bxb1 without NLS (*p* = 0.007). SV40 NLS and EGL-13 NLS also moderately enhanced nSIE of Bxb1 integrase as N-terminal (*p* = 0.0004) or C-terminal fusion (*p* = 0.002), respectively (Fig. 2A). The least effective NLS was c-Myc NLS, with neither N- nor C-terminal fusion enhancing the nSIE of Bxb1 integrase (Fig. 2A).

In addition to natural NLS sequences, we also generated a rationally designed artificial NLS sequence based on the data generated by Kosugi et al. [21] to see if the optimized amino acid usage of NLS sequence would increase the stable integration efficiency. The rational design of the NLS was implemented by introducing the most optimal amino acid in terms of NLS activity to each position of the nucleoplasmin NLS sequence. However, the rationally designed NLS produced an average nSIE of 1.29, considerably lower than that of Bxb1-NPLC. Given the significance of positively charged amino acids for NLS sequence function [19], we investigated the correlation between the number of such amino acids in each NLS sequence and the corresponding nSIE values. As expected, there was a strong and significant positive correlation between the two variables (Spearman correlation, *r* = 0.7963, *p* = 0.0143, *n* = 9). Furthermore, we computationally determined the net charge of each NLS at pH 7.5 using SnapGene software. The net charges for SV40 NLS, c-Myc NLS, EGL-13 NLS and NPL NLS were 4.61, 1.61, 4.61 and 7.61, respectively. Assessing the correlation between the positive net charge and nSIE, a strong and statistically significant correlation was observed (Spearman correlation, *r* = 0.9223, *p* = 0.0011, *n* = 9). In fact, the correlation between the net charge and nSIE was stronger and more significant than the correlation between the number of positively charged amino acids and nSIE.

### Stable integration efficiency was further enhanced by adding two nucleoplasmin nuclear localization signals to the C-terminus of Bxb1 integrase

**Fig. 3:**
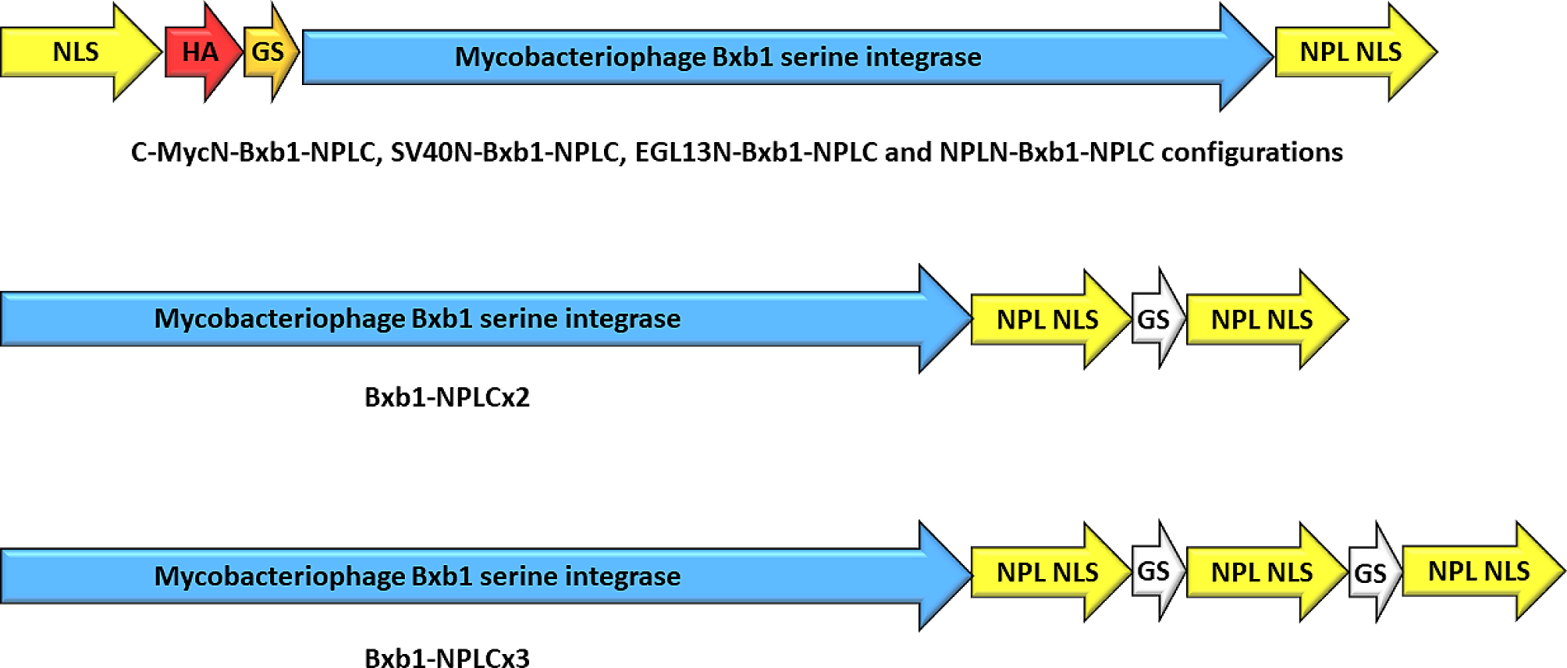
Double and triple NLS variants of Bxb1 integrase. Construct on top shows the configration of the double NLS variants with NLSs at N- and C-terminus. Constructs in the middle and at the bottom illusrate the configuration of Bxb1-NPLCx2 and Bxb1-NPLCx3 variants, respectively. NLS sequences of Bxb1-NPLCx2 and Bxb1-NPLCx3 are separated by a flexible GGGGSGGGGSGS linker (white arrow)

After evaluating the Bxb1–NLS variants with a single NLS fusion at the N- or C-terminus, we aimed to further enhance the nSIE by incorporating additional NLS sequences into the Bxb1 integrase. Given that the C-terminal fusion with NPL NLS demonstrated the most significant improvement among the tested variants compared to the Bxb1 without an NLS fusion, we focused on this variant for further optimization. To this end, we cloned the SV40 NLS, c-Myc NLS, EGL-13 NLS and NPL NLS to the N-terminus of Bxb1–NPLC variant. Furthermore, we generated Bxb1–NLS variants with two and three NPL NLS sequences, each separated by a 12-residue Gly/Ser-rich linker, at the C-terminus of the Bxb1 integrase (Fig. 3). The double and triple NLS variants were named SV40N–Bxb1–NPLC, c-MycN–Bxb1–NPLC, EGL-13 N–Bxb1–NPLC, NPLN–Bxb1–NPLC, Bxb1–NPLCx2 and Bxb1–NPLCx3. These variants were subsequently assessed for their impact on stable integration efficiency as before.

**Fig. 4:**
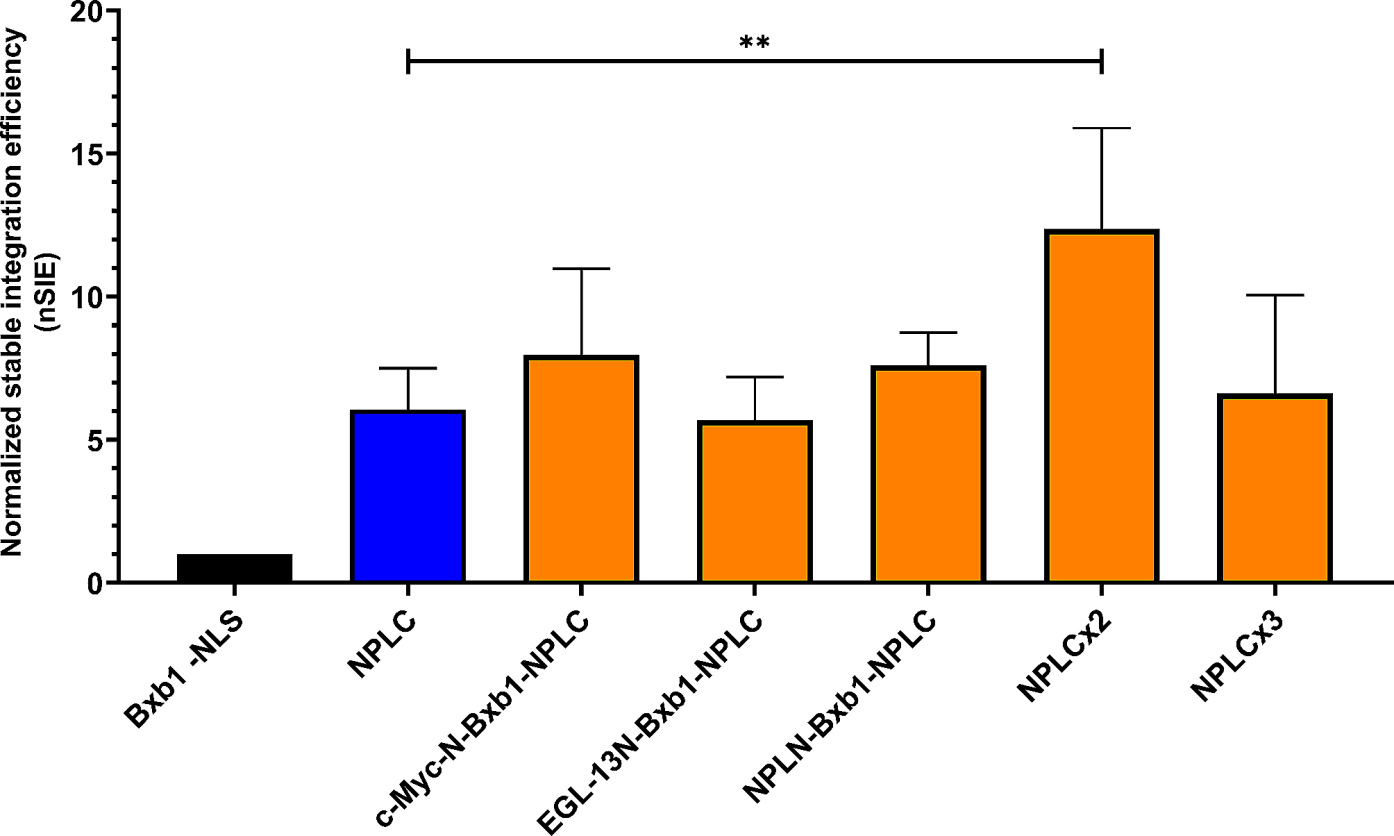
Comparison of the best single NLS variant, Bxb1-NPLC, and Bxb1–NLS variants containing two or three NLS sequences. The bars represent the average normalized stable integration efficiencies (nSIE) with error bars representing the standard deviation of three independent experiments (except NPLC, *n* = 6; NPLCx2, *n* = 9).(**) *p* < 0.01

The results, depicted in Fig. 4, presents the average nSIE values of the best single NLS variant, Bxb1-NPLC, and the double/triple NLS variants. When compared to Bxb1–NPLC, the double NLS variant Bxb1–NPLCx2 significantly increased nSIE of Bxb1 integrase twofold (*p* = 0.0012). None of the other double or triple NLS variants showed significant increase in nSIE compared to Bxb1–NPLC, but in general all the double NLS variants had significantly higher nSIE values than most of the single NLS variants. Furthermore, the average nSIE of all the double and triple NLS variants was threefold higher than that of single NLS variants (*p* = 1.2 × 10^− 9^). Interestingly, addition of the third NPL NLS sequences to the C-terminus of the Bxb1 integrase (Bxb1–NPLCx3) didn’t further increase the nSIE but caused a slight decrease in the average nSIE. The performance of the Bxb1–NPLCx3 variant showed a significant difference when compared to only one single NLS variant, namely Bxb1–c-MycC.

### Co-expression of Ran GTPase-activating protein 1 (RanGAP) with Bxb1-NPLCx2 serine integrase increased stable integration efficiency

**Fig. 5:**
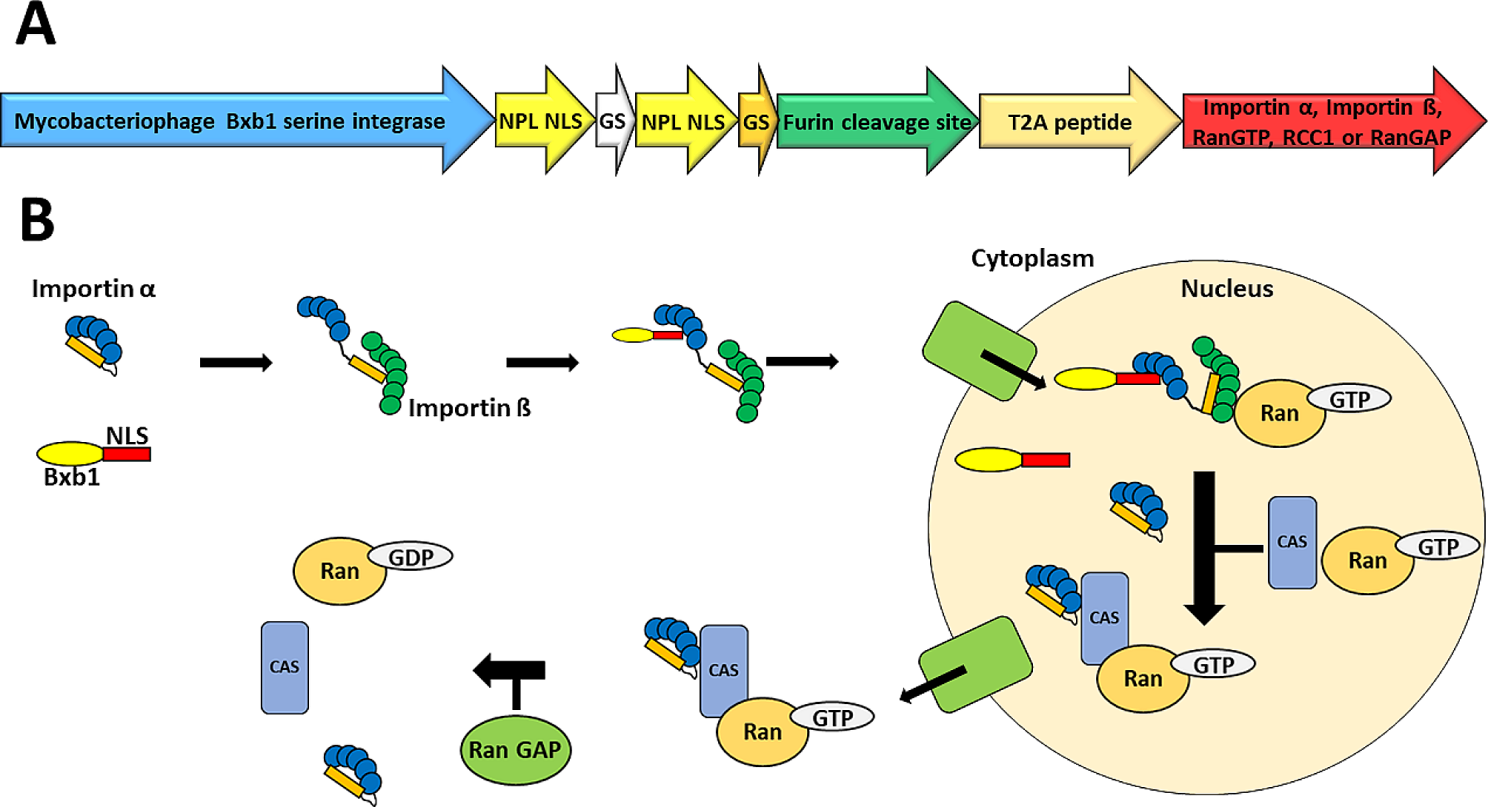
Co-expression of proteins involved in the translocation of proteins into the nucleus. **(A)** Schematic illustration of the genetic construct expressing both Bxb1–NPLCx2 and Importin α, Importin ß, RanGTP, RCC1 or RanGAP. The two proteins were separated by GS linker, furin clevage site and T2A peptide. **(B)** Illustration of the translocation process of proteins into the nucleus [20]. Importin α interacts with importin ß through IBB (Importin-ß-binding) domain (orange block), exposing the binding cavity of importin α, allowing the binding of NLS sequence to it. The ternary complex is then translocated into the nucleus where binding of RanGTP to importin ß causes dissociation of the ternary complex. Subsequently, importin α binds to exportin complex composed of CAS and RanGTP, and the complex is transported out from the nucleus. The exportin complex is dissociated in the cytoplasm by Ran GAP, converting Ran-GTP to RanGDP. Ran-GDP is converted back to the active Ran-GTP form in the nucleus by regulator of chromosome condensation 1 (RCC1) protein (not shown in the figure)

In addition to evaluating different NLS sequences and their combinations, we hypothesized that the amount and availability of certain proteins involved in the nuclear translocation process could be the bottleneck limiting the nuclear transport and thus the integration efficiency. To test our hypothesis, we overexpressed proteins involved in the nuclear translocation process to assess their impact on the stable integration efficiency. Specifically, mouse importin α, importin ß, GTP-binding nuclear protein Ran (RanGTP), regulator of chromosome condensation 1 (RCC1) and Ran GTPase-activating protein 1 (RanGAP) were co-expressed with the Bxb1–NPLCx2 variant. These transport proteins were expressed from the same plasmid as the Bxb1–NPLCx2, under the same promoter. The genes were separated from the Bxb1-NPLCx2 by a GS linker, furin cleavage site and T2A self-cleaving peptide, enabling individual expression of each protein (Fig. 5A). The roles of importin α, importin ß, RanGTP, RCC1 and RanGAP in the nuclear translocation process are illustrated in Fig. 5B.

**Fig. 6:**
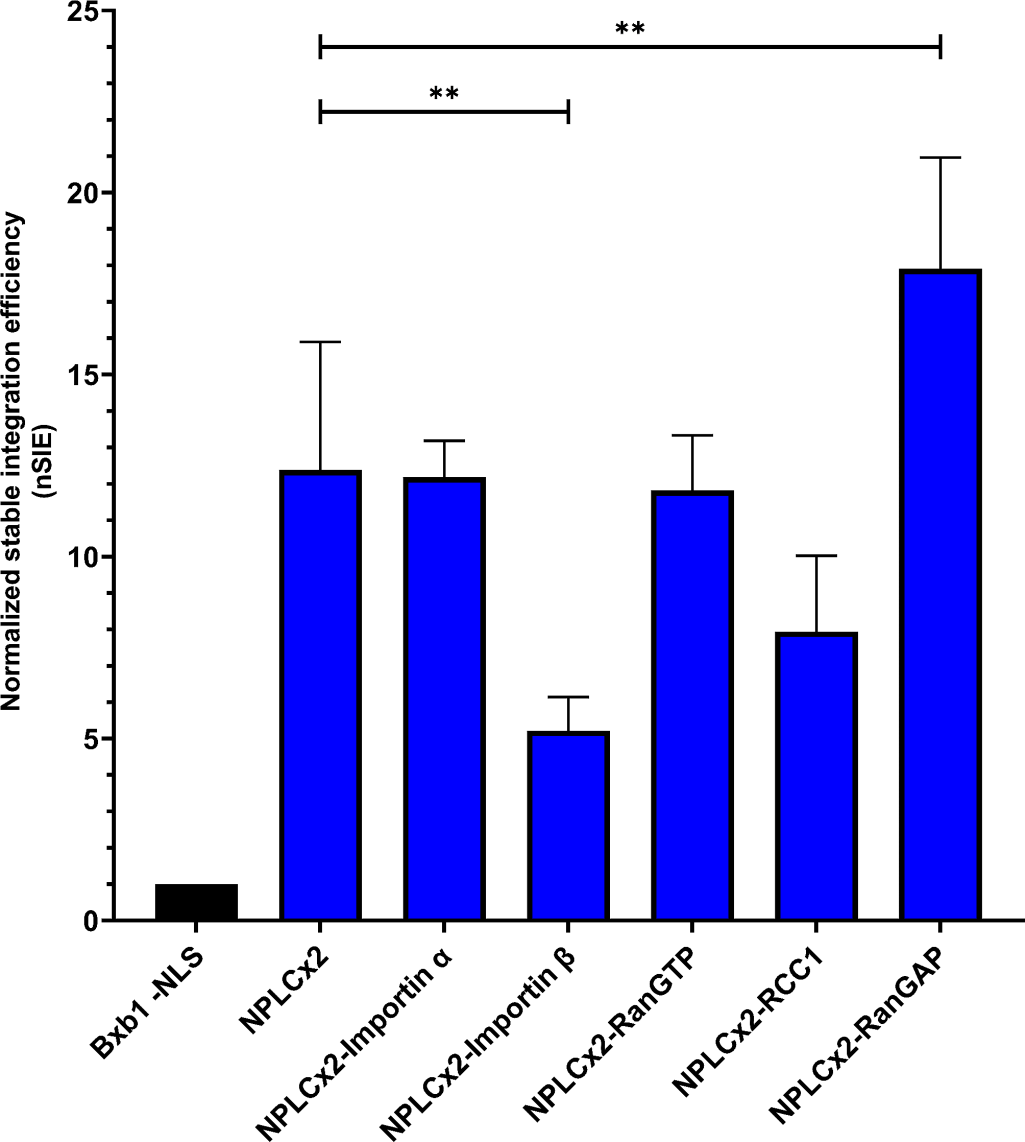
Effect of co-expression of nuclear transport proteins. The graph shows the average normalized stable integration efficiencies (nSIE) of each Bxb1–NPLCx2-nuclear transport protein construct. The error bars represent standard deviation of three replicate transfections (except NPLCx2, *n* = 9; NPLCx2-RanGAP, *n* = 6). Asterisks denote statistically significant difference to NPLCx2. (**) *p* < 0.01

The effect of co-expressing the transport proteins on the stable integration efficiency was assessed as before. Of all the tested transport proteins, RanGAP was the only one showing statistically significant positive effects on stable integration efficiency (Fig. 6). The co-expression of RanGAP with Bxb1-NPLCx2 increased the stable integration efficiency by 45% compared to Bxb1-NPLCx2 expression alone. The rest of the co-expressed transport proteins had either insignificant or negative effects on stable integration efficiency. For example, co-expression of importin β and RCC1 lowered stable integration efficiency by 58% and 36%, respectively, whereas RanGTP and importin α didn’t have a significant effect.

### Improved integration efficiency was also applicable to antibody genes

**Fig. 7:**
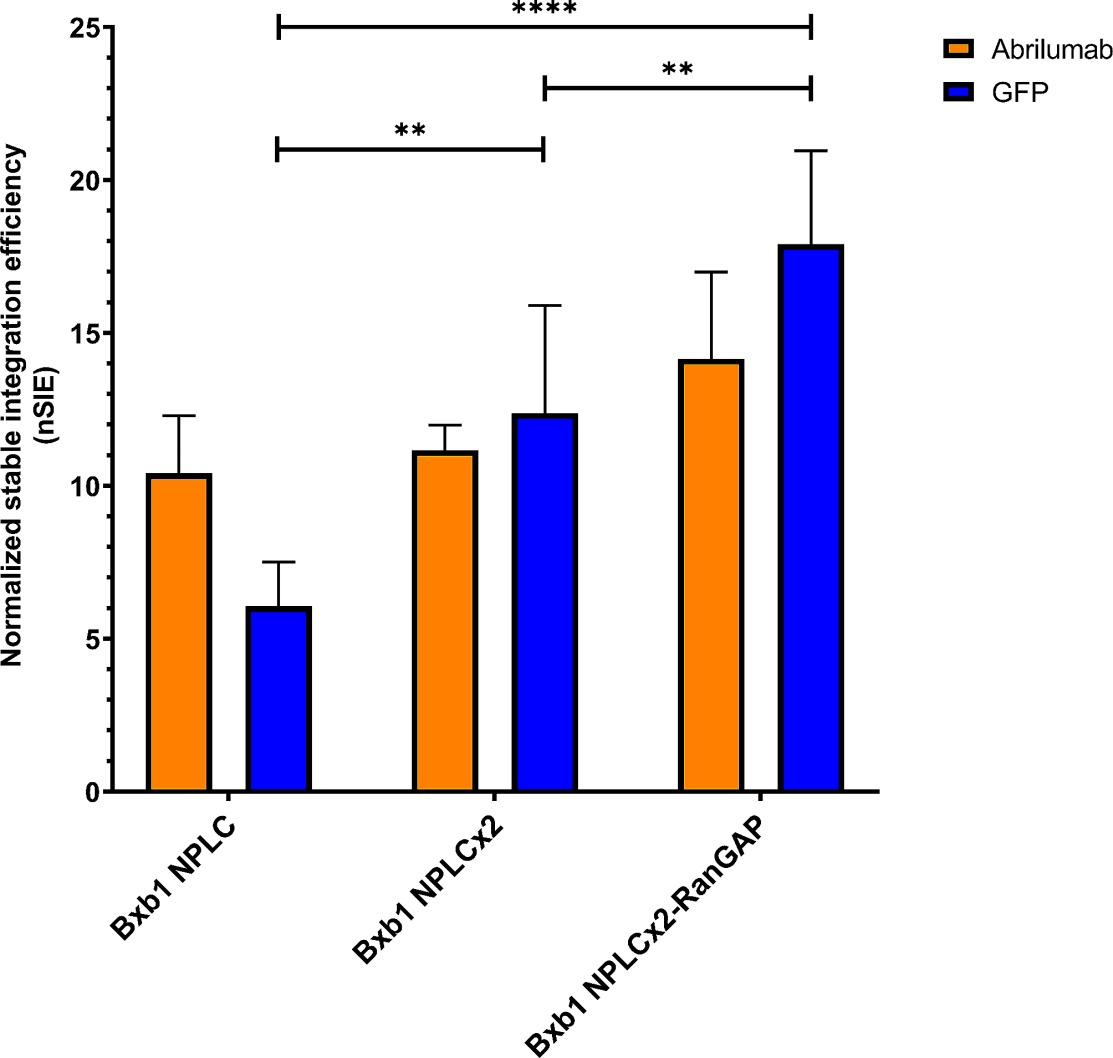
Comparison of the normalized stable integration efficiency of Bxb1-NPLC, Bxb1-NPLCx2 with and without RanGAP co-expression using abrilumab (orange) and GFP (blue). The stable integration efficiencies are normalized to Bxb1 without NLS fusion. The error bars represent standard deviation of three replicate transfections (abrilumab) or six replicate transfections (GFP, except Bxb1-NPLCx2 *n* = 9). (**) *p* < 0.01; (***) *p* < 0.001

We aimed to validate that the observed findings, obtained with the relatively small and easily expressed GFP protein, could be applied to more complex multimeric protein constructs, such as antibodies. In pursuit of this, we conducted a comparative analysis between Bxb1 without NLS, Bxb1-NPLC, Bxb1-NPLCx2, and Bxb1-NPLCx2 co-expressed with RanGAP, using a DNA construct designed for surface display of the clinical antibody abrilumab. The nSIE of abrilumab showed over a 10-fold increase using the Bxb1-NPLC, 11-fold increase using the Bxb1-NPLCx2 and a 14-fold increase with NPLCx2 with co-expression of RanGAP, compared to Bxb1 without NLS (Fig. 7). The nSIE of abrilumab was marginally improved by 7% with the inclusion of two copies of the NPL in the C-terminus, compared to having one copy. The co-expression of RanGAP also slightly improved the nSIE of abrilumab by 27% compared to the Bxb1-NPLCx2 variant. Although the differences between the different NLS variants were more modest when using abrilumab compared to GFP, the magnitude of the improvement over Bxb1 lacking NLS was similar regardless of the size of the integrated construct. These findings indicate that the enhanced stable integration efficiency is also applicable to larger gene constructs such as antibodies.

## Discussion

Bxb1 integrase has been extensively used for the stable genomic integration of protein variant libraries into mammalian cells [7, 8, 10, 11, 23, 24]. The likelihood of finding a protein variant with desired properties rises with the expansion of the variant library size. Engineering parts of the Bxb1 integrase-mediated genomic integration process is needed since the library size is governed by the genomic integration efficiency. This study investigates the impact of various NLS types and overexpression of proteins involved in the nuclear translocation process on Bxb1 integrase-mediated stable integration efficiency in CHO cells harboring a landing pad for the stable genomic integration of genes of interest.

First, we tested how different NLS types and various NLS fusion configurations affect Bxb1 integrase-mediated stable integration efficiency. We observed that nucleoplasmin NLS showed a significant increase in the stable integration efficiency as both N- and C-terminal fusions to Bxb1 integrase. The most significant effect was observed when NLS was introduced as a C-terminal fusion to Bxb1 integrase, resulting in over a sixfold increase in stable integration efficiency compared to Bxb1 integrase without NLS. To the best of our knowledge, SV40 NLS fusion is used in all the published studies describing the use of Bxb1 integrase for genomic integration in mammalian cells [7–11, 25]. Noteworthy, our study showed that the stable integration efficiency with nucleoplasmin NLS fusion was also substantially better than that of the SV40 NLS. Furthermore, nucleoplasmin NLS was the only NLS that increased stable integration efficiency as both fusion configurations. For comparison, the least effective NLS, c-Myc NLS, did not increase stable integration efficiency neither as N- nor C-terminal fusion to Bxb1 integrase.

As the genomic integration catalyzed by Bxb1 integrase occurs in the nucleus, the increased stable integration efficiency seen with the NLS variants was likely attributed to the enhanced nuclear localization of Bxb1 integrase. This is in line with the findings by Fang et al., who demonstrated more efficient nuclear localization of GFP with nucleoplasmin NLS fusion compared to GFP with c-Myc NLS fusion in *Phytophthora sojae* [18]. On the contrary, Ray et al. reported opposite outcomes in HeLa cells, indicating that the efficiencies of NLS sequences may differ based on the organisms [26]. Beyond the host organism, the properties of the translocated protein also influence the selection of an optimal NLS [25]. In summary, identifying the most effective NLS sequence for a given protein and host organism may necessitate a careful and comprehensive testing and optimization process.

Certain proteins inherently possess two distinct nuclear localization signals each contributing to nuclear transport [27]. In our experiments, we observed a significant increase in stable integration efficiency when two nucleoplasmin NLS sequences were added to the C-terminus of Bxb1 integrase. The inclusion of three nucleoplasmin NLS sequences did not result in a further increase in stable integration efficiency; instead, it unexpectedly decreased it (Fig. 4). In previous work, Goldfarb et al. found that the rate of nuclear translocation process is saturable [28]. This observation may account for the lack of a higher stable integration efficiency with three nucleoplasmin NLS sequences compared to two in our study.

Co-expression of nuclear transport proteins exhibited diverse effects on stable integration efficiency. For instance, co-expression of RanGAP with Bxb1-NPLCx2 increased the stable integration efficiency by 45%, while co-expression of importin β substantially decreased it. Previous studies have shown that RanGAP overexpression enhances NLS-mediated nuclear transport of DNA cargo [29], aligning with our results. Importantly, as demonstrated by the stable integration of genes encoding membrane-anchored antibody abrilumab, these findings are not limited to small genes, such as those encoding monomeric proteins like GFP, but also extend to much larger genetic constructs, including antibody genes and libraries.

The primary motivation for this research was to enhance stable integration to facilitate the construction of larger antibody libraries. In our experiments, the average percentage of cells expressing abrilumab when using Bxb1 lacking the NLS fusion was 0.23%. This value was increased to 3.19% with the inclusion of NPLCx2 fusion and co-expression of RanGAP. This would roughly translate to an antibody library size of 3 × 10^5^ variants per T75 flask (assuming 1 × 10^7^ cells per T75 flask at the time of transfection). Additionally, we have previously adapted the CHO-LP cell line to suspension culture, enabling the cultivation and transfection of a larger number of cells [11], thus allowing the screening of antibody libraries of up to 10^7^ variants. Moreover, although not included in this report focusing on the effect of the NLS fusions, we have also previously enhanced the stable integration efficiency of the Bxb1-SV40N variant by various means including the optimization of the pBxb1-EV to pBxb1-TV plasmid ratio and the total DNA amount as well as by employing electroporation for more efficient transfection [11]. We anticipate that by performing similar optimizations using the Bxb1-NPLCx2-RanGAP variant, it is possible to further improve the stable integration efficiency to accommodate the transfection of even larger antibody libraries.

## Conclusions

In summary, it can be concluded that by optimizing the type of NLS fusion for Bxb1 integrase, the stable integration efficiency can be increased substantially. The study findings can streamline the creation of libraries for mammalian cell display initiatives by employing stable integration of genes into a genomic landing pad.

## Data Availability

All data generated or analyzed during this study are included in this published article.

## Abbreviations

NLS: Nuclear localization signal
CHO: Chinese hamster ovary cell
GFP: Green fluorescent protein
RanGAP: Ran GTPase-activating protein
RMCE: Recombinase-mediated cassette exchange
NPC: Nuclear pore complexes
IBB: Importin-β-binding domain
RCC1: Regulator of chromosome condensation 1
LP: Landing pad
CMV: Cytomegalovirus
BSD: Blasticidin S deaminase
c-Myc: Myc proto-oncogene
SV40: Simian virus 40 large T antigen
EGL-13: Transcription factor EGL-13
NPL: Nucleoplasmin from *Xenopus laevis*
nSIE: Normalized stable integration efficiency
RanGTP: GTP-binding nuclear protein Ran
